# Zeolitic Imidazolate Frameworks as Zn^2+^ Modulation Layers to Enable Dendrite‐Free Zn Anodes

**DOI:** 10.1002/advs.202002173

**Published:** 2020-08-09

**Authors:** Xiaoqing Liu, Fan Yang, Wei Xu, Yinxiang Zeng, Jinjun He, Xihong Lu

**Affiliations:** ^1^ MOE of the Key Laboratory of Bioinorganic and Synthetic Chemistry The Key Lab of Low‐Carbon Chem and Energy Conservation of Guangdong Province School of Chemistry Sun Yat‐Sen University Guangzhou 510275 P. R. China; ^2^ School of Applied Physics and Materials Wuyi University Jiangmen Guangdong 529020 P. R. China

**Keywords:** ion modulation, zeolitic imidazolate frameworks, zinc dendrites anodes, Zn anodes, Zn‐based batteries

## Abstract

Zinc (Zn) holds great promise as a desirable anode material for next‐generation rechargeable batteries. However, the uncontrollable dendrite growth and low coulombic efficiency of the Zn plating/stripping process severely impede further practical applications of Zn‐based batteries. Here, these roadblocks are removed by using in situ grown zeolitic imidazolate framework‐8 (ZIF‐8) as the ion modulation layer to tune the diffusion behavior of Zn^2+^ ions on Zn anodes. The well‐ordered nanochannels and N species of ZIF‐8 can effectively homogenize Zn^2+^ flux distribution and modulate the plating/stripping rate, ensuring uniform Zn deposition without dendrite growth. The Zn corrosion and hydrogen evolution are also alleviated by the insulating nature of ZIF‐8, resulting in high coulombic efficiency. Therefore, the Zn@ZIF anode shows highly reversible, dendrite‐free Zn plating/stripping behavior under a broad range of current densities, and a symmetric cell using this anode can work correctly up to 1200 h with a low polarization at 2 mA cm^−2^. Moreover, this ultrastable Zn@ZIF anode also enables a full Zn ion battery with outstanding cyclic stability (10 000 cycles).

## Graphical abstract

Zeolitic imidazolate framework‐8 (ZIF‐8) is in situ grown on Zn surface as Zn^2+^ modulation layers to achieve a dendrite‐free Zn plating behavior. Benefiting from the well‐ordered nanochannels and abundant N species, the ZIF‐8 can effectively homogenize Zn^2+^ flux and moderate plating/stripping rate, contributing to a high‐efficiency and long‐life Zn@ZIF anode.

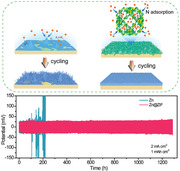

Metallic Zn is regarded as a promising anode for aqueous batteries due to its numerous inherent advantages of high abundance, nontoxicity, easy processing, high theoretical gravimetric and volumetric capacities (820 mAh g^−1^ and 5855 mAh cm^−3^), as well as low electrochemical potential (−0.76 V vs standard hydrogen electrode).^[^ ^1^ ^]^ Stimulated by these distinct merits, a number of aqueous rechargeable Zn‐based batteries including Zn//MnO_2_,^[^ ^2^ ^]^ Zn//V_2_O_5_,^[^ ^3^ ^]^ Zn//I_2_,^[^ ^4^ ^]^ and Zn//Co batteries^[^ ^5^ ^]^ have been developed and demonstrated encouraging performance. However, the two major detrimental issues that Zn anodes persistently suffer from restrict further applications of these energy storage devices: 1) the inhomogeneous Zn plating/stripping is very likely to trigger the growth of Zn dendrites, which may puncture the separator and cause internal short circuit.^[^ ^6^ ^]^ 2) Zn dendrite growth is frequently accompanied by competitive hydrogen evolution, which limits the coulombic efficiency (CE) of Zn anode. Beyond that, large amount of OH^−^ ions left by water decomposition would lead to Zn corrosion and passivation, which further lower the utilization efficiency and cyclability of Zn anodes.^[^ ^7^ ^]^ Therefore, the further breakthrough of aqueous Zn‐based batteries lies in the development of dendrite‐free Zn anodes allowing highly reversible Zn plating/stripping.

In recent years, extensive efforts have been devoted to address the aforementioned issues and multifarious approaches have been proposed to achieve dendrite‐free Zn anode, such as constructing 3D conductive hosts^[^ ^8^ ^]^ and artificial protective films,^[^ ^9^ ^]^ using concentrated electrolyte,^[^ ^10^ ^]^ introducing additives into the electrolyte,^[^ ^11^ ^]^ etc. Very recently, metal‐organic frameworks (MOFs) are enabled as functional materials to regulate the diffusion, nucleation, and deposition behaviors of Zn^2+^, for the sake of achieving highly reversible Zn plating/stripping with dendrite‐free morphology.^[^ ^12^ ^]^ For instance, by utilizing a MOF annealed at 500 °C as a desirable host to guide uniform Zn deposition, Wang et al. reported a symmetric cell with a stable CE of 98.6% over 200 cycles at 2 mA cm^−2^.^[^ ^12a^ ^]^ Pitifully, the preparation process of the Zn‐MOF‐500 anode includes a 12 h MOF growth, high temperature calcination, slurry coating and Zn plating, which seems too complicated for repeated tests or mass production. To simplify the synthesis route, Yuksel et al. precoated the Zn anode with an artificial layer of ZnO to induce the formation of an ≈400 µm MOF coverage.^[^ ^12b^ ^]^ The cycling performance of such MOF‐integrated electrode was better than bare Zn but still utterly disappointing, which might be attributed to the insulating nature of the thick MOF and the incomplete etching of ZnO precursor. Further derivation of MOFs to N‐doped porous carbons by virtue of high‐temperature pyrolysis endowed the Zn electrode with favorable cycling durability up to 400 h at 2 mA cm^−2^. However, the basic frameworks and unique structure of MOFs were hardly preserved after pyrolysis and the electrochemical properties of MOF‐based Zn anodes are still far from satisfactory. Briefly, although MOFs with specific porosity feature hold great promise for the development of efficient Zn anodes with long lifespan, to date, it is still impossible to achieve satisfactory energy storage performance by directly using MOFs without further treatment.

Different from previous reports, herein, to achieve a high‐efficiency and long‐life anode, in situ grown thin zeolitic imidazolate framework‐8 (ZIF‐8) layer on commercial Zn foil (Zn@ZIF), prepared by a one‐step solvent thermal method, is designed to be an effective ion modulation layers to tune the diffusion behaviors of Zn^2+^ ions. Characterized by well‐ordered porous channels and abundant N species, ZIF‐8 can effectively harmonize Zn^2+^ flux and modulate the plating rate at the interface of Zn anode. Specifically, the high energy barrier of ZIF‐8 makes the diffusion of Zn^2+^ ions on Zn@ZIF‐8 not as free as that on bare Zn, thereby effectively avoiding the tip‐effect‐induced Zn dendrite growth and boosting the stability of Zn anode. At the same time, the insulating feature of ZIF‐8 enables the layer to function as an inert physical barrier to alleviate Zn corrosion and H_2_ evolution, contributing to higher CE of Zn plating/stripping. Based on this versatility, the symmetric cell based on Zn@ZIF anode exhibits exceptional cyclicality for over 1200 h with small voltage hysteresis of ≈58 mV at 2 mA cm^−2^, showing a nearly ninefold increment in lifetime compared with bare Zn. This Zn@ZIF anode also delivers a high average CE of 99.4% at 5 mA cm^−2^ and 5 mAh cm^−2^. More importantly, a rechargeable Zn ion battery is fabricated based on the Zn@ZIF anode, which presents exceptionally long lifespan with no capacity attenuation for 10 000 cycles.

The ZIF‐8 was in situ grown on Zn foil through a facile solvent thermal procedure utilizing Zn foil as the Zn source (Experimental Section). The 3D frameworks topologies of ZIFs reveal that metal Zn centers are regularly coordinated to four imidazolate ligands, forming abundant inner cavities and large channels (**Figure** **1**a). Notably, the imidazolate ligands also offer rich N source for adsorption of Zn ion. Scanning electron microscopy (SEM) images show that the surface of Zn foil is homogenously covered by microparticles with irregular polyhedral morphology (Figure 1b) while the surface of bare Zn foil is relatively smooth without particles (Figure S1, Supporting Information). From the cross‐section SEM image in Figure 1c, the thickness of the ZIF layer on the surface of Zn foil is about 1 µm. The corresponding energy‐dispersive spectroscopy (EDS) elemental mapping images confirm that ZIF‐8 with obvious existence of C and N is mainly observed in the surface layer (Figure 1d). To further explore the detailed information of the Zn@ZIF sample, the atomic force microscopy (AFM) was conducted to monitor its 3D morphology (Figure 1e). Compared with the relatively smooth surface of Zn foil (Figure S2, Supporting Information), the surface layer of Zn@ZIF sample is uneven with randomly distributed microparticles, consistent with SEM results.

**Figure 1:**
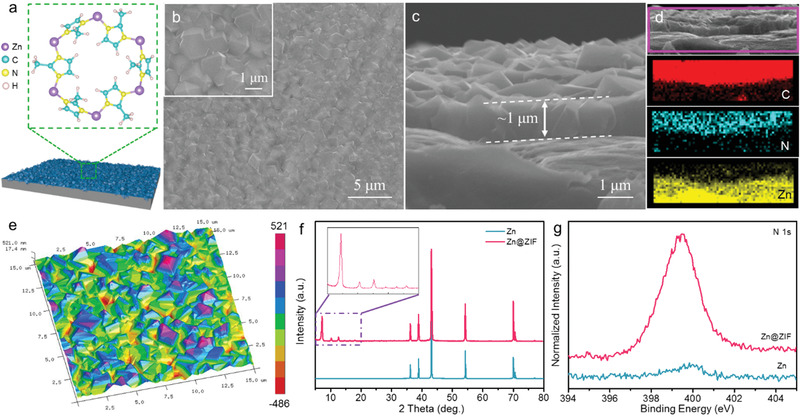
a) The schematic diagrams for the Zn@ZIF sample; b) SEM images; c) cross‐section SEM image; d) EDS elemental mapping images; e) AFM 3D morphology of the Zn@ZIF sample; f) XRD patterns; g) N 1s XPS spectra of the bare Zn and Zn@ZIF samples.

The chemical composition of the Zn and Zn@ZIF samples was further studied by X‐ray diffraction (XRD) and X‐ray photoelectron spectroscopy (XPS) analysis. As shown in Figure 1f, except for the typical diffraction peaks assigned to Zn (JCPDS no. 87‐0713), the Zn@ZIF sample displays obvious peaks matched well with the previously reported ZIF‐8, indicating that Zn foil could act as the sacrifice Zn^2+^ source to form ZIF‐8 structure with high crystallinity. Moreover, the N 1s XPS spectra of the Zn and Zn@ZIF samples in Figure 1g further verify the existence of N in Zn@ZIF sample. The peak for N 1s located at 399.5 eV can be attributed to the N connected with C in imidazole rings.^[^ ^13^ ^]^ The Zn 2p spectra of the Zn can be deconvoluted into two peaks corresponding to Zn 2p_1/2_ and Zn 2p_3/2_ of Zn^0^. Both peaks for Zn@ZIF sample shifted to higher angel, which might be ascribed to the fact that the metal Zn center is Zn^2+^ in the ZIF‐8 (Figure S3, Supporting Information). All these results comprehensively demonstrate that a layer of ZIF‐8 microparticles is uniformly coated on the surface of Zn foil.

To investigate the influence of ZIF layer on Zn deposition morphology, ex situ SEM characterizations were conducted. When the deposition current density and capacity are set at 1 mA cm^−2^ and 1 mAh cm^−2^, respectively, the bare Zn exhibits a bumpy surface with some protuberance and Zn nanosheets (**Figure** **2**a). As the current density increasing to 2 mA cm^−2^ with a fixed plating capacity of 2 mAh cm^−2^, the moss‐like Zn agglomeration grows into tens of micronometers with laminated surface (Figure 2b). The cross‐section SEM image in Figure 2c revealed that the thickness of Zn agglomeration is as high as 18 µm. With the further increment of current density to 5 mA cm^−2^, the Zn protrusions become even larger (Figure S4a, Supporting Information), suggesting the uncontrolled and tip‐induced proliferation of Zn dendrites on bare Zn. Note that when the deposition capacity is set to 2 mAh cm^−2^, a higher plating current density might induce larger protrusions (Figure S4b,c, Supporting Information). In sharp contrast, the surface of Zn@ZIF electrode is homogenous with nanosheets morphology at various current densities (1, 2, and 5 mA cm^−2^) and there is no uneven protuberance detected (Figure 2d,e; Figure S4d, Supporting Information). From the cross‐section SEM image (Figure 2f), the surface of the Zn@ZIF electrode is smooth and the thickness of deposited Zn is about 6 µm with a total capacity of 2 mAh cm^−2^, indicative of the uniform and compact Zn deposition.

**Figure 2:**
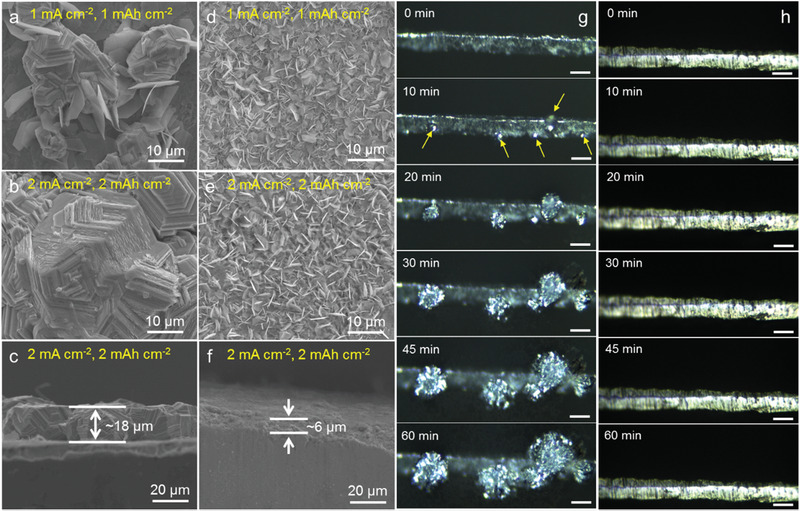
a–f) SEM images and cross‐section SEM images of bare Zn and Zn@ZIF electrodes after plating Zn at different current densities with various capacities. The optical photographs at the Zn/electrolyte interface during Zn plating process recorded at different times for g) bare Zn and h) Zn@ZIF electrodes.

Furthermore, the macroscopic morphological evolution of Zn deposition process was in situ visualized by optical microscopy imaging using a specially designed cell device. Here, a transparent electrochemical cell consisting of two Zn metal electrodes and ZnSO_4_ electrolyte was fabricated and the symmetric cell was subject to a high plating current density of 5 mA cm^−2^. The optical images of the Zn/electrolyte interface were real‐time acquired at different stages during Zn plating process (Figure 2g,h). As displayed in Figure 2g, a number of visible spots appeared after deposition for 10 min. With the increase of the deposition time, the growth of protuberance boomingly evolved and triggered a self‐amplification behavior. After 1 h, the uncontrolled Zn dendrites with large size (>50 µm) finally spread on the Zn anode surface, which might result in the potential risks of short‐circuit or other safety hazards. In comparison, the Zn@ZIF electrode showed a smooth surface during the whole plating process, and no visible dendrites was observed (Figure 2h). Both the SEM observation and optical microscopy tracking manifest that the porous ZIF‐8 layer can effectively suppress the formation of Zn dendrites.

To evaluate the cycling stability of Zn metal anodes, galvanostatic cycling performance of symmetric Zn cells was tested at various current densities and capacities. **Figure** **3**a compares the cycling stability of Zn and Zn@ZIF cells at a current density of 2 mA cm^−2^ with a capacity limitation of 1 mA h cm^−2^. The bare Zn|Zn cell exhibited random voltage oscillations and a sudden voltage increase after 130 h, which might be derived from the dynamic dendrite‐induced soft short circuit. In contrast, the cell based on Zn@ZIF electrode presented a stable polarization voltage (≈58 mV) and an ultralong cycling life over 1200 h, which prolonged the lifespan of Zn anode by nearly ninefolds. The corresponding magnified view of the voltage profiles of cells with bare Zn and Zn@ZIF electrodes in inset of Figure 3a showed that both bare Zn and Zn@ZIF electrodes gave a steady voltage plateau at 30th cycles. With further cycling, the Zn@ZIF electrode exhibited an obvious preponderance than bare Zn electrode. As the capacity is extended to 2 mA h cm^−2^, the Zn@ZIF cell can still maintain good stability without apparent overpotential increment for 700 h, while the bare Zn|Zn cell failed at about 100 h (Figure 3b). In addition, when charged–discharged at a high current density of 5 mA cm^−2^ for 0.5 or 1 h, the Zn@ZIF cell can operate stably up to 900 or 400 cycles, respectively, suggesting superior durability of the Zn@ZIF anode (Figure S5, Supporting Information). Such outstanding cycling performance also outperforms most recently reported Zn anodes.^[^ ^8^, ^9^, ^11^, ^12^, ^14^ ^]^

**Figure 3:**
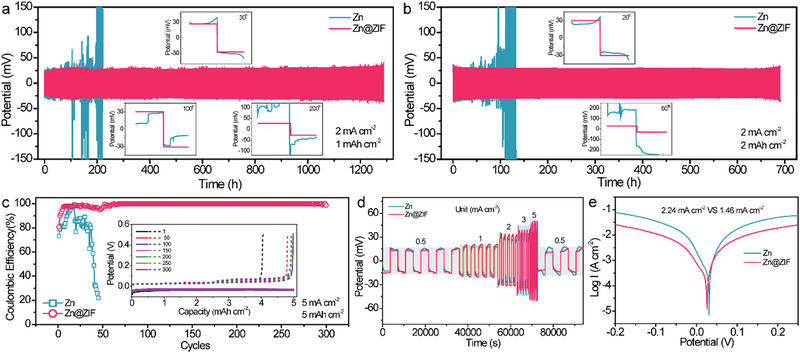
Voltage profiles of symmetric cells based on bare Zn foil and Zn@ZIF anodes at a) 2 mA cm^−2^ with a capacity of 1 mAh cm^−2^ and b) 2 mA cm^−2^ with a capacity of 2 mAh cm^−2^. c) CE of the Zn plating/stripping on bare Zn and Zn@ZIF anodes at 5 mA cm^−2^ with a capacity of 5 mAh cm^−2^. Inset shows the corresponding Zn plating/stripping curves after certain cycles. d) Rate performance of the Zn and Zn@ZIF anodes during continuous cycling at current densities increasing from 0.5 to 5 mA cm^−2^ with a constant capacity of 0.5 mAh cm^−2^. e) Corrosion curves of the Zn and Zn@ZIF anodes.

The morphology and XRD patterns of the Zn and Zn@ZIF electrodes after long‐term cycling were also investigated to shed more light on the cycling process (Figure S6, Supporting Information). Identical fluffy and mossy structure with long and disordered acicular Zn was observed on the surface of bare Zn, suggesting the uncontrolled Zn dendrite growth. It is noted that the large protrusions (about 80 µm) and the needle‐like Zn dendrites are prone to penetrate separator, causing short circuit of battery. By contrast, the Zn@ZIF equipped with Zn^2+^ modulation layer displays a compact, smooth surface without any protruding filaments. Figure S7 (Supporting Information) presents the XRD patterns of the Zn and Zn@ZIF electrodes after cycling. Apart from the strong peaks of Zn (JCPDS no. 87‐0713), the remaining peaks for both electrodes can all be assigned to Zn_4_SO_4_(OH)_6_⋅5H_2_O (JCPDS no. 39‐0688), which presumably stems from the side reaction of ZnSO_4_ electrolyte.^[^ ^15^ ^]^ Compared with bare Zn, the Zn@ZIF electrode exhibited lower peaks for Zn_4_SO_4_(OH)_6_⋅5H_2_O, signifying its capability to suppress side reactions of electrolyte. In addition, the electrical impedance spectrum (EIS) characterizations for Zn and Zn@ZIF symmetry cells after various cycles were conducted. As shown in Figure S8 (Supporting Information), the charge transfer resistance (*R*_ct_) for both Zn|Zn and Zn@ZIF|Zn@ZIF cells was similar at first 25 cycles. But the Zn symmetric cell exhibited a sharp increase in resistance (>1000 Ω) from 25 to 50 cycles, which might be ascribed to the formation of Zn dendrite and consumption of electrolyte, consequently resulting in cell failure.

CE during Zn plating/stripping is an essential parameter to evaluate the reversibility of the Zn metal anode. Here, a cell based on Zn or Zn@ZIF anode and carbon fiber cathode was adopted to disclose the cycling efficiency of Zn anode. The CE of cell with pristine Zn anode decayed rapidly after only 30 cycles at 5 mA cm^−2^ with a capacity of 5 mAh cm^−2^. In contrast, an average CE of 99.4% after 300 cycles can be achieved by the cell with Zn@ZIF anode, which is believed to be originated from the dendrite‐free plating process induced by the porous ZIF‐8 coverage (Figure 3c). The corresponding stable Zn plating/stripping curves with small voltage fluctuation and hysteresis further verified stable and reversible cycling efficiency of the Zn@ZIF anode. Additionally, the CE measured at different plating current densities and capacities (2 and 5 mA cm^−2^; 2 and 2.5 mAh cm^−2^) drew the same conclusion (Figure S9, Supporting Information). The rate performance of the Zn and Zn@ZIF based symmetric cells was tested by cycling at a spectrum of current densities with a constant capacity of 0.5 mAh cm^−2^ (Figure 3d). The voltage hysteresis of Zn@ZIF was slightly larger than that of bare Zn at high current densities, owing to the fact that the coating of the ZIF‐8 layer slightly increased the interfacial resistance of the Zn symmetrical cell (Figure S10, Supporting Information). After 25 cycles, the Zn@ZIF showed a priority over Zn in voltage hysteresis, delivering a better cyclic stability. To gain insight into the beneficial effect of the porous ZIF layer, we measured the Zn corrosion of Zn and Zn@ZIF electrodes by linear polarization first. As shown in Figure 3e, the corrosion current density of Zn@ZIF electrode (1.46 mA cm^−2^) is much lower than that of bare Zn electrode (2.24 mA cm^−2^), indicating a lower corrosion rate of Zn with ZIF coating. Furthermore, we compared the H_2_ evolution behaviors of Zn and Zn@ZIF electrode using two electrode system. As shown in Figure S11 (Supporting Information), the H_2_ evolution is easier to take place on bare Zn, indicating that the ZIF‐8 protective layers could alleviate the H_2_ evolution reaction to some extent. This might be attributed to the insulating feature of ZIF‐8. The restriction of H_2_ evolution and Zn corrosion both contributes to the high CE of Zn@ZIF.

To probe the influence of porous ZIF layer on the Zn deposition process, Zn adsorption and diffusion were investigated by density functional theoretical (DFT) calculation. Note that Zn metal is used the reference for all the theoretical calculations. First, we perform first‐principles calculation for the binding energies of Zn^2+^ on Zn (001) and ZIF‐8 (Figure S12, Supporting Information). Compared with Zn (001), ZIF‐8 has much strong interaction with Zn^2+^ (Figure S13, Supporting Information). Specifically, there is only a weak interaction between Zn^2+^ and Zn (001) with binding energy of −0.62 eV (**Figure** **4**a) while the ZIF‐8 shows a higher binding energy of −1.22 eV with N as adsorption site, suggesting the strong interaction between Zn^2+^ and N atoms (Figure 4b). Figure 4c displays charge density and differential charge density of the most stable configurations for ZIF‐8 with Zn^2+^ adsorption. In this way, the ZIF‐8 layer can adhere and immobilize a certain amount of Zn^2+^ during Zn plating process, thereby alleviating the concentration gradient at the interface. Such unique characteristic is capable of effectively avoiding the tip‐effect‐induced Zn dendrite growth observed on bare Zn. We also calculated the diffusion barrier of Zn^2+^ to migrate from one energy minima to the other nearby minima on Zn (001) and ZIF‐8 (Figure S14, Supporting Information). The activation energy for Zn on Zn (001) surface is only 0.02 eV, revealing that Zn diffusion on the Zn (001) surface is essentially instantaneous, which favors inhomogeneous mossy‐like Zn deposition (Figure 4d).^[^ ^16^ ^]^ In contrast, ZIF‐8 presents a considerably higher activation energy of 1.38 eV. Such high energy barrier for Zn^2+^ diffusion of ZIF‐8 plays a substantially important role in guiding dendrite‐free Zn deposition on Zn@ZIF. As we know, at the nucleation stage, a pivotal step for homogeneous Zn plating, there exist inevitably a certain number of preferential nucleation sites on the anode surface and the initial deposition of Zn will always take place on these sites. If the diffusion barrier is too low, Zn^2+^ ions are prone to continuously diffuse onto these Zn nuclei and the resultant tip effect will lead to the growth of Zn dendrites. That is the reason why we see the occurrence of Zn dendrites on bare Zn anodes. In contrast, the high Zn^2+^ diffusion barrier (−1.38 eV) is capable of impeding the migration and agglomeration of Zn^2+^ ions to some extent, thus functioning as a buffer or modulating layer to homogenize the Zn^2+^ ion flux or slow down the diffusion rate of Zn^2+^. The combination of high adsorption energy and surface diffusion barrier would endow the Zn@ZIF‐8 anode with the capability to getting rid of the dendrite interference.

**Figure 4:**
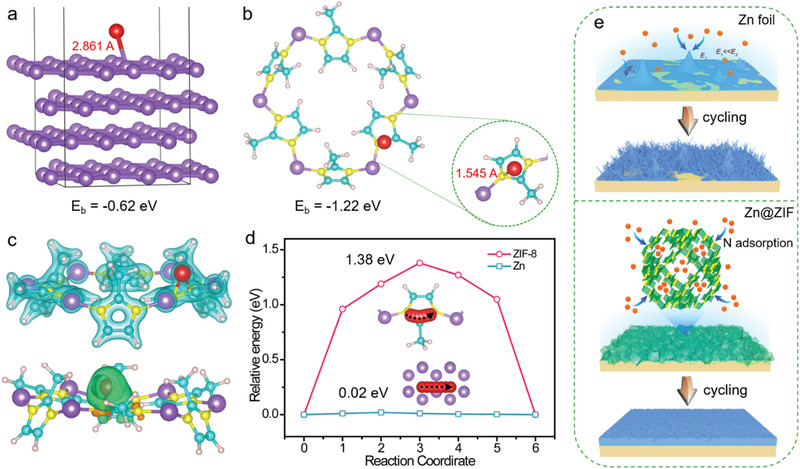
The calculated binding energy of Zn^2+^ on a) Zn (001) and b) ZIF‐8. c) Charge density diagram and charge density differences of the ZIF with Zn^2+^ adsorption. d) The activation energy for Zn^2+^ to migrate from one energy minima to the other nearby minima on Zn (001) and ZIF. e) The schematic diagrams for the bare Zn and Zn@ZIF anodes during cycling.

Based on the above results, the proposed schematic illustrations of Zn plating on Zn and Zn@ZIF are graphically depicted in Figure 4e. For bare Zn anode, Zn^2+^ prefer to accumulate at early formed small Zn deposits due to a relatively shorter diffusion pathway and stronger electric field. With the continuous cycling under long‐time and high deposition capacity condition, such tip effect proliferates at the electrolyte–Zn interface, causing severe random ramified Zn growth and significantly degraded lifespan. With the presence of porous ZIF layer, the ordered nanosized channels in ZIF‐8 can divide the space above Zn anode into small confinements, Zn^2+^ flux in each confined nanochannel could be comparatively homogeneous, guiding a uniform Zn nucleation and plating rate. Moreover, benefiting from the strong interaction between Zn^2+^ ions and N atoms of ZIF‐8, a certain amount of Zn^2+^ will be held by N species. Therefore, when the Zn^2+^ ions in the close vicinity of the Zn surface are consumed during the plating course, the supplementary of Zn^2+^ ions would be more effective on Zn@ZIF‐8, thus ensuring more homogeneous Zn deposition.

To further verify the superiority of Zn@ZIF anode in practical Zn ion batteries, the Zn@ZIF anode was paired with a LaVO_4_ cathode. For this, the LaVO_4_ cathode was prepared according to a previous research and it showed a nanofibers structure (Figure S15a, Supporting Information).^[^ ^17^ ^]^ All the XRD peaks are in good accordance with the LaVO_4_ (JCPDS no. 50‐0367), proving no other impurities (Figure S15b, Supporting Information). And there is a peak at low angle of 6.8° corresponding to an interlayer spacing of 13.1 Å, which is conducive to the intercalation of Zn^2+^. **Figure** **5**a compares the CV profiles of Zn ion batteries with Zn and Zn@ZIF anodes. Both curves show two pairs of distinct redox peaks with similar current density, verifying that the ZIF layer has negligible effect on the reaction of Zn ion battery. Identical conclusion can be obtained in studies where these two cells with Zn and Zn@ZIF anodes display similar galvanostatic charge/discharge curves at different current densities (Figure 5b; Figure S16, Supporting Information). To highlight, the highest capacity of this LaVO_4_//Zn@ZIF battery reached 255 mAh g^−1^ at 0.5 mA cm^−2^, yielding an energy density of 209 Wh kg^−1^ (Figure 5c). As the current density increased to 10 mA cm^−2^, a high capacity of 84.6 mAh g^−1^ was still maintained, accompanied with a decent capacity retention of 33.2% (Figure S17, Supporting Information).

**Figure 5:**
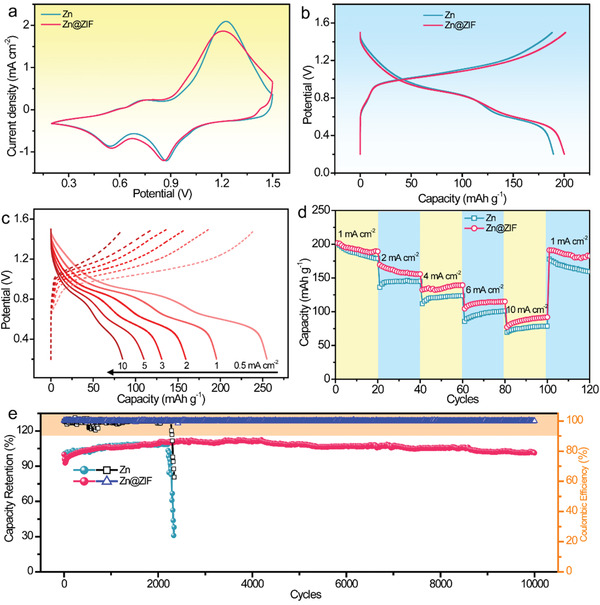
a) CV curves at 0.5 mV s^−1^; b) charge–discharge profiles at 1 mA cm^−2^ of the Zn ion batteries with Zn and Zn@ZIF anodes; c) charge–discharge curves of the LaVO_4_//Zn@ZIF battery at different current densities; d) rate performance at various current densities; e) cycling performance and CE tested at 10 mA cm^−2^ for 10 000 cycles of the LaVO_4_//Zn and LaVO_4_//Zn@ZIF batteries.

Rate performance and long‐term cycling stability of the Zn ion batteries were also studied to unravel the influence of ZIF coating on Zn anode. As shown in Figure 5d, the Zn ion batteries with Zn and Zn@ZIF anode delivered nearly the same capacity (≈200 mAh g^−1^) at 1 mA cm^−2^. However, the capacity difference is growing larger with further cycling under current densities of 2, 4, 6, 10 mA cm^−2^. When resetting back to 1 mA cm^−2^, the LaVO_4_//Zn@ZIF battery recovered to a considerable average capacity of 185 mAh g^−1^, higher than that of bare LaVO_4_//Zn battery (166 mAh g^−1^). More importantly, the superior durability of Zn@ZIF anode stemming from effective suppression of dendrite growth also promises Zn ion batteries with long lifespans. Figure 5e presents the long‐term cycling performance of Zn ion batteries based on Zn and Zn@ZIF anode for 10 000 cycles. An excellent cyclic stability with capacity retention of 101% was achieved by the LaVO_4_//Zn@ZIF battery, accompanied with an average CE of 99.8%. Yet, with bare Zn anode, the Zn ion battery showed sharp capacity attenuation after only 2270 cycles.

In conclusion, we proposed an in situ grown porous ZIF‐8 as an ideal Zn^2+^ modulation layer to effectively regulate the aqueous Zn deposition behavior. The well‐organized nanosized channels in ZIF‐8, combined with its insulating feature, could guide homogeneous Zn^2+^ flux onto the Zn/electrolyte interface and get rid of the side reactions, ensuring uniform Zn nucleation and deposition with high efficiency. DFT calculations revealed that the N species of ZIF‐8 endow the Zn@ZIF‐8 anode with high adsorption energy and high surface diffusion barrier for Zn^2+^ ions, both contributing to the efficient elimination of potential tip effect, as well as the resultant dendrite interference. With these synergistic effects, the symmetric Zn@ZIF|Zn@ZIF cell displayed stable polarization voltage (≈58 mV) and ultralong cyclic stability for over 1200 h with dendrite‐free morphology. In contrast, the symmetric cell with bare Zn anode could survive for only 130 h with fluffy and mossy Zn dendrite, indicating that ZIF can significantly suppress Zn dendrites growth and promote the lifespan of Zn anode. Furthermore, when paired Zn@ZIF anode with LaVO_4_ cathode, this assembled Zn ion battery exhibited impressive cycling performance with 101% capacity retention after 10 000 cycles, which is much better than battery with bare Zn anode and other reported Zn ion batteries. The concept of employing porous ZIFs as ion modulation layer could offer valuable insights for the design of dendrite‐free Zn anode, which brings new possibilities for realizing ultrastable Zn‐based batteries.

## Experimental Section

##### Preparation of Zn@ZIF

First, Zn foil (50 µm) was sonicated in ethanol for 15 min, followed by soaking in 0.5 mol L^−1^ HCl solution for 2 min to remove the extra zinc oxides. Then, the washed cleaned Zn foil was used as substrate to grow ZIF and immersed into a solution in a Teflon‐lined stainless‐steel autoclave. The solution for ZIF growth was prepared by dissolving 2 g 2‐methylimidazole in 20 mL methanol. The autoclave was heated at 100 °C for 8 h and naturally cool down to room temperature. The Zn foil was washed with ethanol and dried in vacuum drying oven at 60 °C to obtain the final Zn@ZIF sample.

##### Preparation of LaVO_4_

LaVO_4_ was prepared according to the previous research. Specifically, 0.182 g V_2_O_5_ was dissolved into a mixture of 49 mL distilled water and 1 mL H_2_O_2_, followed by magnetically stirring for 5 h. Then, 0.221 g La(NO_3_)_3_⋅6H_2_O was added to the above solution and stirred for 5 min. This mixed solution was transferred into a Teflon‐lined stainless‐steel autoclave and heated to 120 °C for 6 h. Finally, the product was centrifuged and washed with distilled water and ethanol for three times, and dried in vacuum oven at 60 °C. The LaVO_4_ electrode was prepared by mixing the powder with carbon black, and polyvinylidene fluoride with the weight ratio of 8:1:1, and 1‐methyl‐2‐pyrrolidinone was use as the solvent. The final slurry was coated onto carbon paper and then dried in a vacuum oven at 60 °C for 12 h. The active mass loading of the electrode was 1.9 mg cm^−2^.

##### Material Characterization

The microstructures and compositions of the Zn, Zn@ZIF and LaVO_4_ samples were characterized by X‐ray diffractometry (XRD, D8 ADVANCE), XPS (XPS, Nexsa, Thermo FS), field‐emission SEM (FE‐SEM, JSM‐6330F), and AFM (SPM‐9500J3).

##### Electrochemical Measurement

CV, corrosion test, and galvanostatic charge/discharge were tested using an electrochemical workstation (PARSTAT MC) at room temperature. All the tests were performed in a two‐electrode cell with electrolyte of 2 m ZnSO_4_. To analyze the cycling performance of symmetric cells, 2032‐type coin cells were assembled with Zn foil and Zn@ZIF anodes, glass fiber separator, and 200 *µ*L electrolyte. To investigate the coulombic efficiency of Zn plating/stripping, Zn foil or Zn@ZIF was used as counterelectrode, and carbon cloth was employed as working electrode. The stripping cutoff voltage was set at 0.5 V (vs Zn^2+^/Zn) for each cycle. The H_2_ evolution behavior was tested using two‐electrode system with stainless‐steel foil as working and Zn foil or Zn@ZIF as reference and counterelectrodes.

The specific capacities of the cell were estimated from the discharge curve using the following equations

(1) $$C_{\mathrm{cell}}=\frac{\int_{0}^{\Delta t}I\times dt}{m}$$

where *C*_cell_ (mA h g^−1^) is the specific capacity of the Zn ion battery, *I* (mA) is the applied discharging current, Δ*t* (h) is the discharging time, and *m* (g) is the mass of the LaVO_4_ cathode.

The specific energy density *E* of the cell was obtained by the following equations

(2) $$E=\int_{0}^{\Delta V}C_{\mathrm{cell}}\times \mathrm{dV}$$

where *E* (Wh kg^−1^) is the energy density, *C*_cell_ is the specific capacity obtained from Equation (1), and *V* (V) is the voltage window.

##### DFT Calculation

The first‐principles calculations were performed in the Vienna ab initio simulation package (VASP) based on the density functional theory (DFT). The general gradient approximation (GGA) and Perdew–Burke–Ernzerhof (PBE) exchange‐correlation functions were utilized in the present calculations. The convergence tolerance quality of geometry optimization was set as 1.0 × 10^−5^ au for energy, and all the forces on each atom were smaller than 0.01 eV Å^−1^. The cutoff energy was set 400 eV for the plane‐wave basis set. The *k*‐points for the Brillion zone were selected by Monkhorts–Pack method and set to 1 × 1 × 1 for ZIF‐8 model and 5 × 5 × 1 for Zn (001). The binding energy (*E*_b_) for each model interacting with a Zn ion was defined as

(3) $$E_{b}=E_{\mathrm{total}}-E_{\mathrm{sub}}-E_{\mathrm{Zn}}$$

where *E*_total_ is the total energy of the ZIF‐8/Zn (001) model bound with Zn ion, *E*_sub_ is the energy of the ZIF‐8/Zn (001) model, and *E*_Zn_ is the energy of Zn ion, respectively. To calculate the diffusion paths of Zn ion on the ZIF‐8/Zn (001) model, the climbing image nudged elastic band (CI‐NEB) method was used.

## Conflict of Interest

The authors declare no conflict of interest.

## Supporting information

Supporting Information
