# Supplementary material for: Zeolitic Imidazolate Frameworks as Zn2+ Modulation Layers to Enable Dendrite‐Free Zn Anodes
Source: Adv Sci (Weinh). 2020 Aug 9;7(21):2002173. doi: 10.1002/advs.202002173 (PMC7610278; doi:10.1002/advs.202002173)
Supplement: Supplementary file 1 — Supporting Information [file ADVS-7-2002173-s001.pdf]

© 2020 Wiley-VCH GmbH

ADVANCED SCIENCE
Open Access

Supporting Information

for *Adv. Sci.*, DOI: 10.1002/advs.202002173

Zeolitic Imidazolate Frameworks as Zn^{2+} Modulation Layers to Enable Dendrite-Free Zn Anodes

Xiaoqing Liu, Fan Yang, Wei Xu, Yinxiang Zeng, Jinjun He, and Xihong Lu**

((Supporting Information can be included here using this template))

Copyright WILEY-VCH Verlag GmbH & Co. KGaA, 69469 Weinheim, Germany, 2018.

Supporting Information

Zeolitic Imidazolate Frameworks as Zn^{2+} Modulation Layers to Enable Dendrite-Free Zn Anodes

Xiaoqing Liu, Fan Yang, Wei Xu, Yinxiang Zeng, Jinjun He, Xihong Lu**

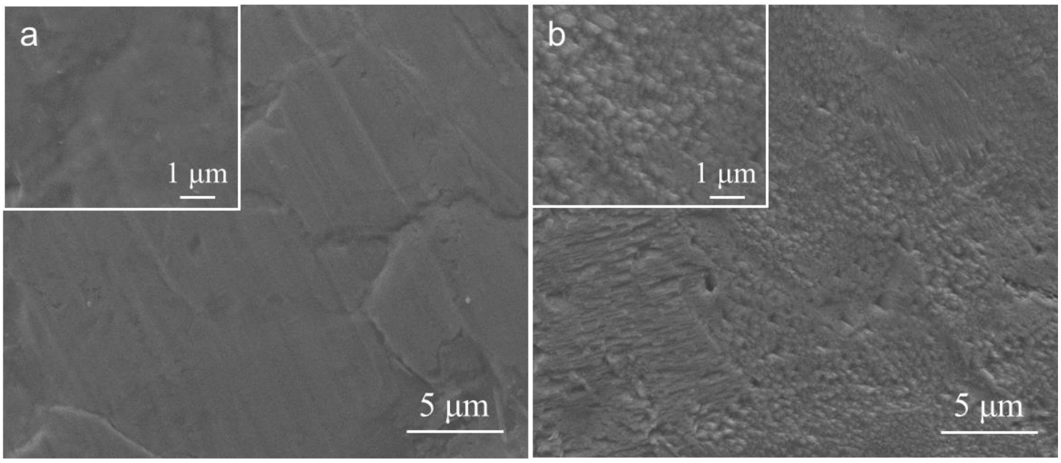

Figure S1. SEM images of the a) commercial Zn foil and b) Zn foil after HCl soaking.

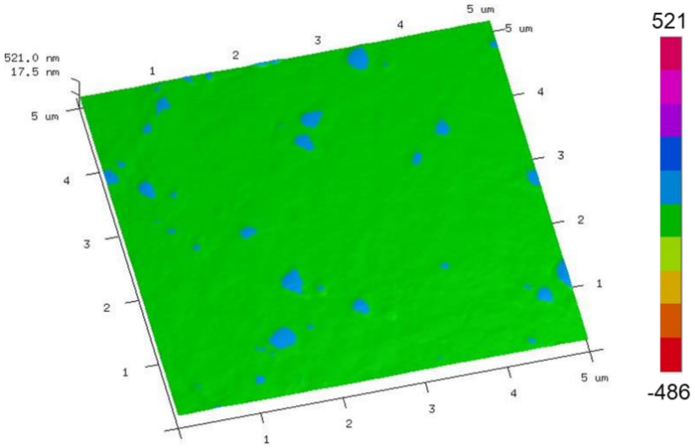

Figure S2. AFM 3D morphology image of the bare Zn sample.

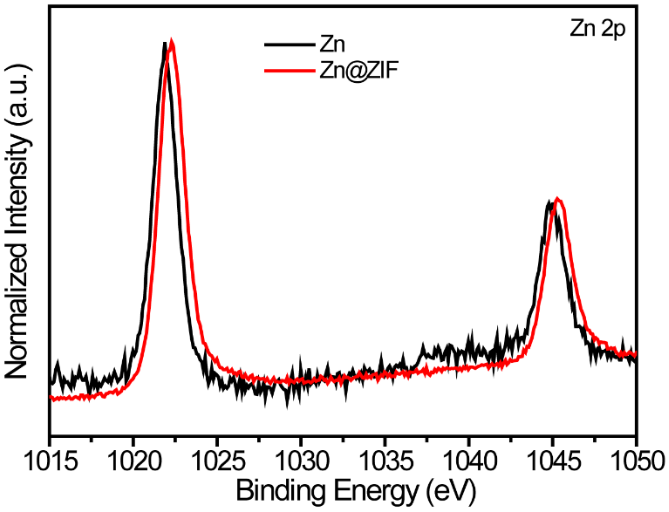

Figure S3. Zn 2p XPS spectra of the bare Zn and Zn@ZIF samples.

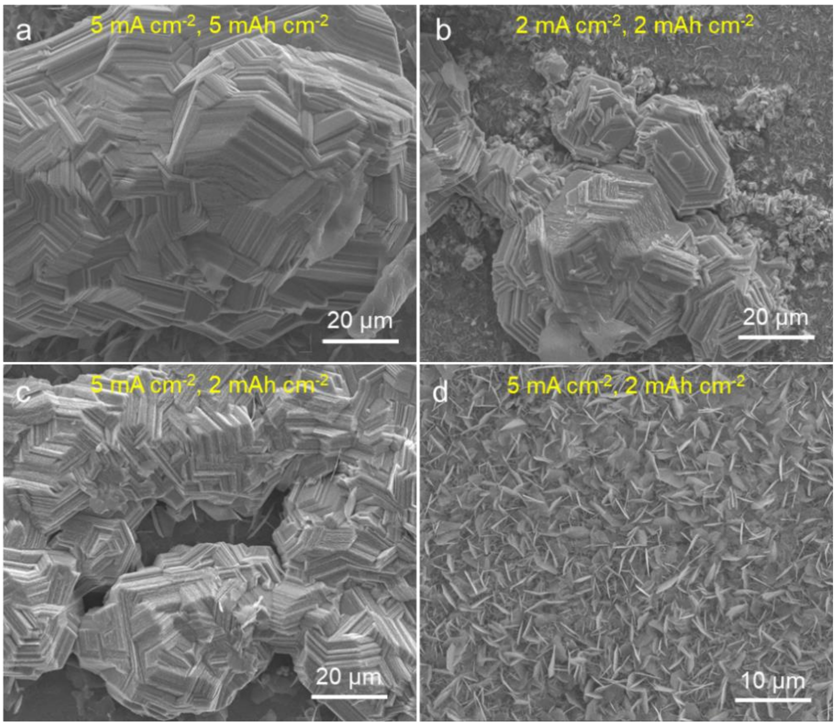

Figure S4. SEM images of the bare Zn electrodes after plating Zn a) at 5 mA cm^{-2} with a capacity of 5 mAh cm^{-2}; b) at 2 mA cm^{-2} with a capacity of 2 mAh cm^{-2}; c) at 5 mA cm^{-2} with a capacity of 2 mAh cm^{-2}. d) SEM image of the Zn@ZIF electrode after plating Zn at 5 mA cm^{-2} with a capacity of 2 mAh cm^{-2}.

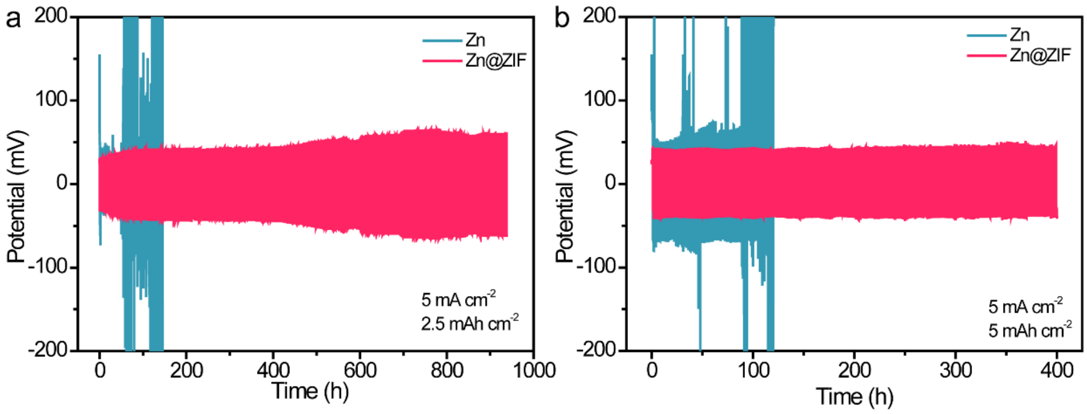

Figure S5. Voltage profiles of symmetric cells based on bare Zn foil and Zn@ZIF anodes at a) 5 mA cm^{-2} with a capacity of 2.5 mAh cm^{-2} and b) 5 mA cm^{-2} with a capacity of 5 mAh cm^{-2}.

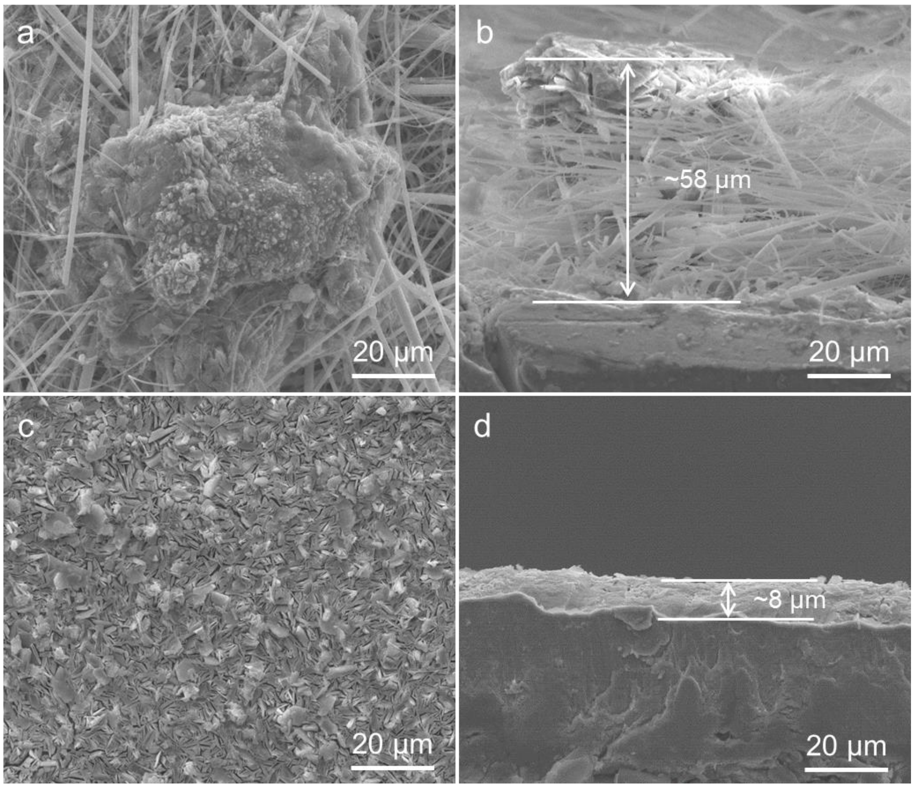

Figure S6. a) SEM image and b) cross-section SEM image of the bare Zn electrode after cycling test. c) SEM image and d) cross-section SEM image of the Zn@ZIF electrode after cycling test.

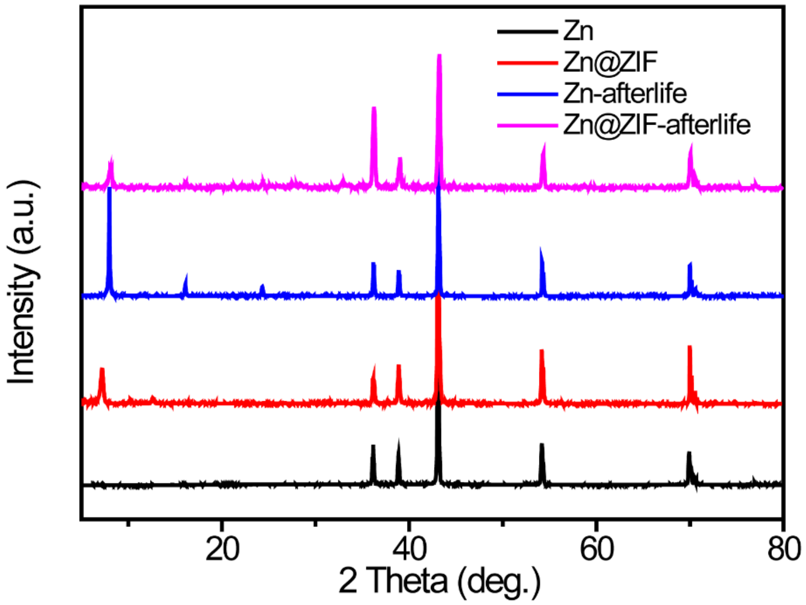

Figure S7. XRD patterns of the bare Zn and Zn@ZIF electrodes before and after cycling test.

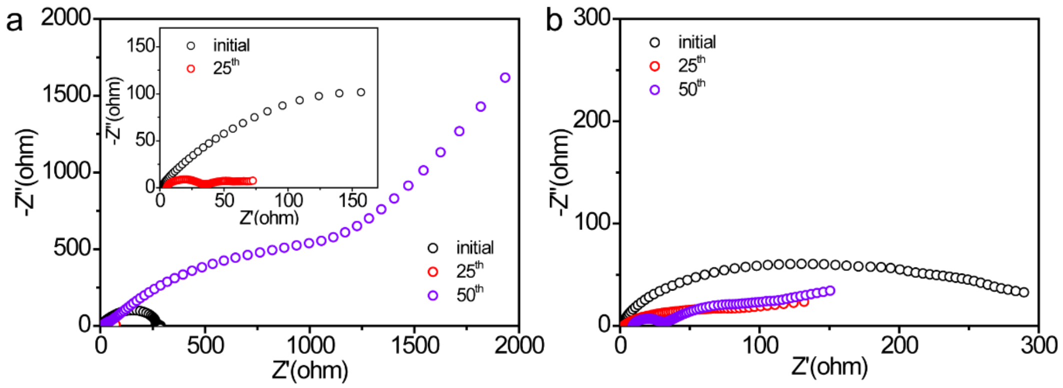

Figure S8. Nyquist plots of the symmetric cells based on a) bare Zn and b) Zn@ZIF electrodes after certain cycles.

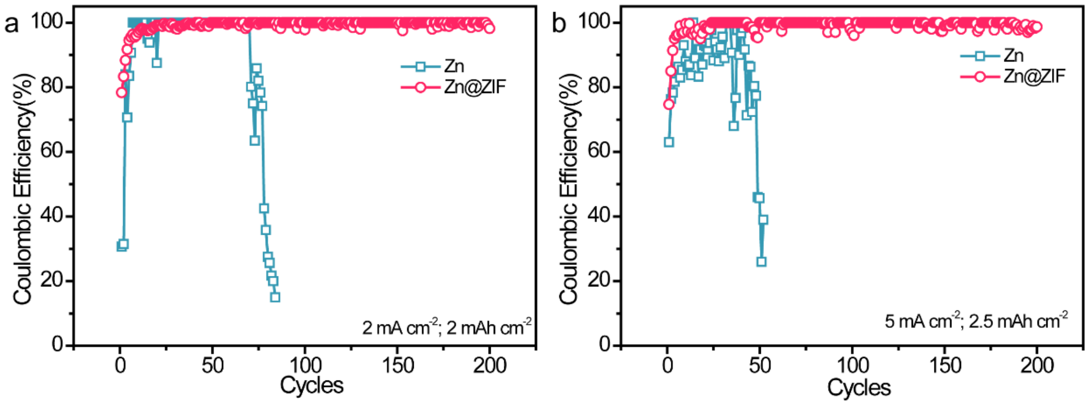

Figure S9. Coulombic efficiencies of the Zn plating/stripping on bare Zn and Zn@ZIF electrodes at a) 2 mA cm^{-2} with a capacity of 2 mAh cm^{-2} and b) 5 mA cm^{-2} with a capacity of 2.5 mAh cm^{-2}.

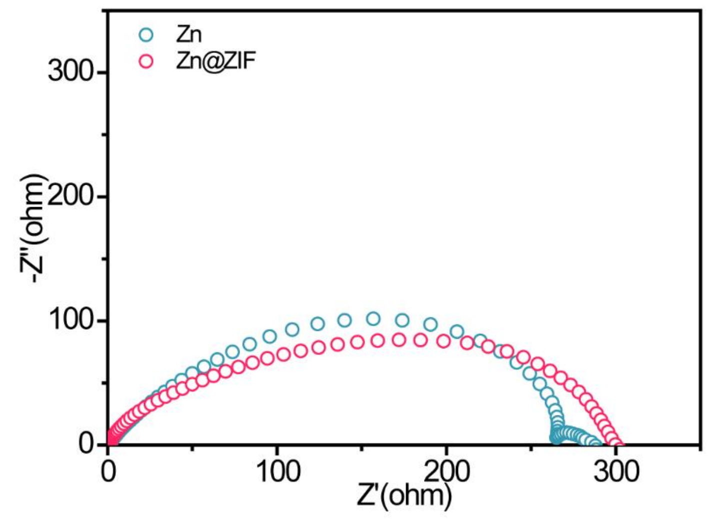

Figure S10. Nyquist plot of the symmetric cells based on bare Zn and Zn@ZIF electrodes.

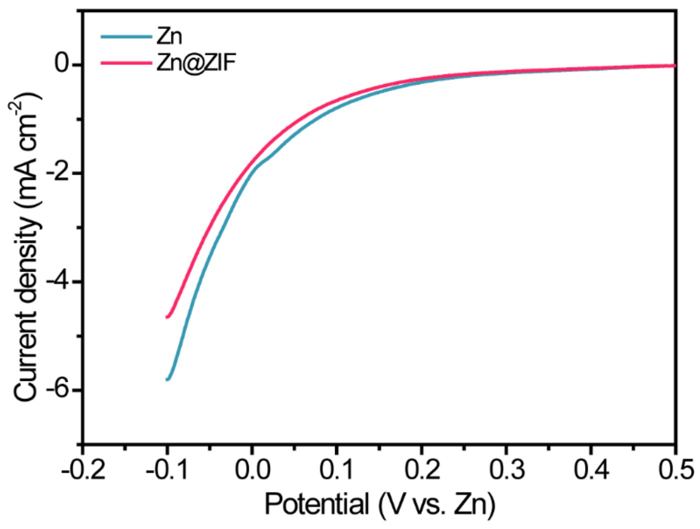

Figure S11 The H_2 evolution behaviors of Zn and Zn@ZIF electrode

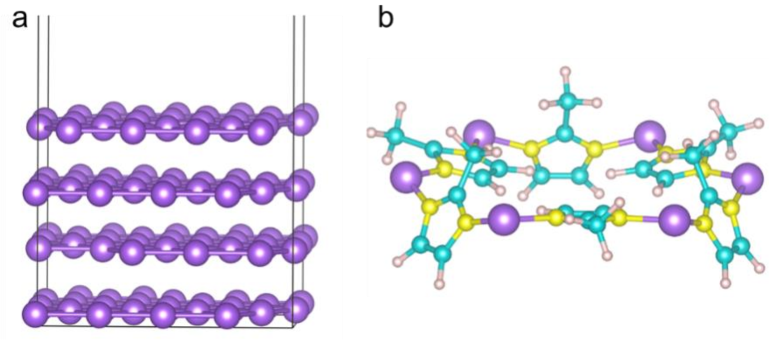

Figure S12. Constructed models of a) Zn (001) and b) ZIF.

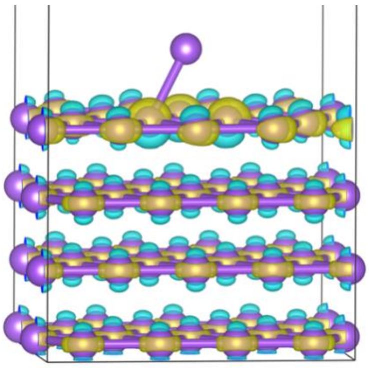

Figure S13. Charge density differences of the Zn (001) with Zn^{2+} adsorption, blue indicates positive charge and yellow indicates negative charge.

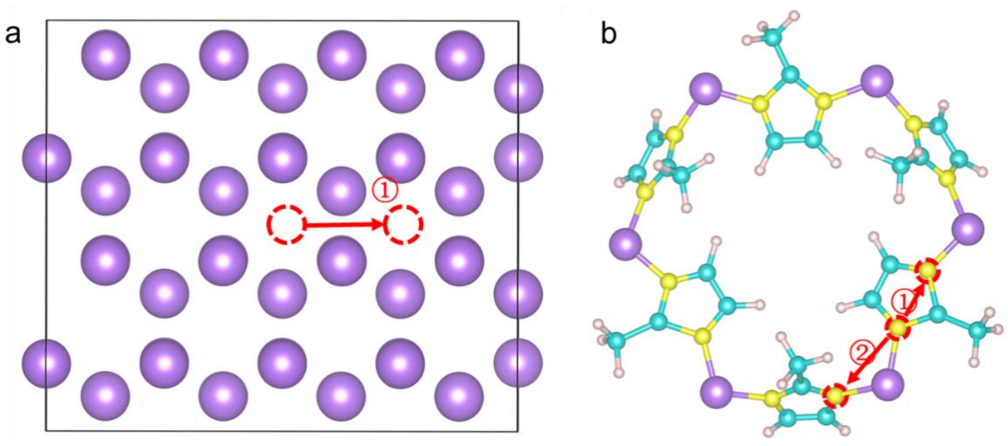

Figure S14. Diffusion paths for Zn^{2+} to migrate from one energy minima to the other nearby minima on a) Zn (001) and b) ZIF surface.

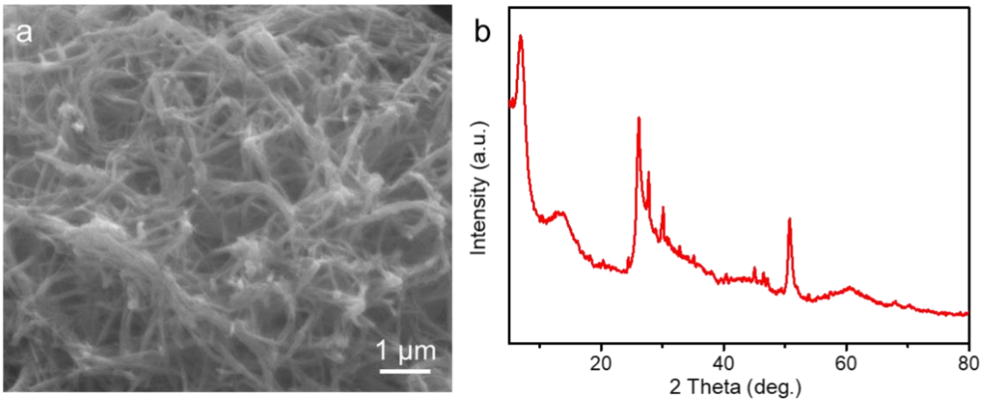

Figure S15. a) SEM images and b) Raman spectrum of the $LaVO_4$ sample.

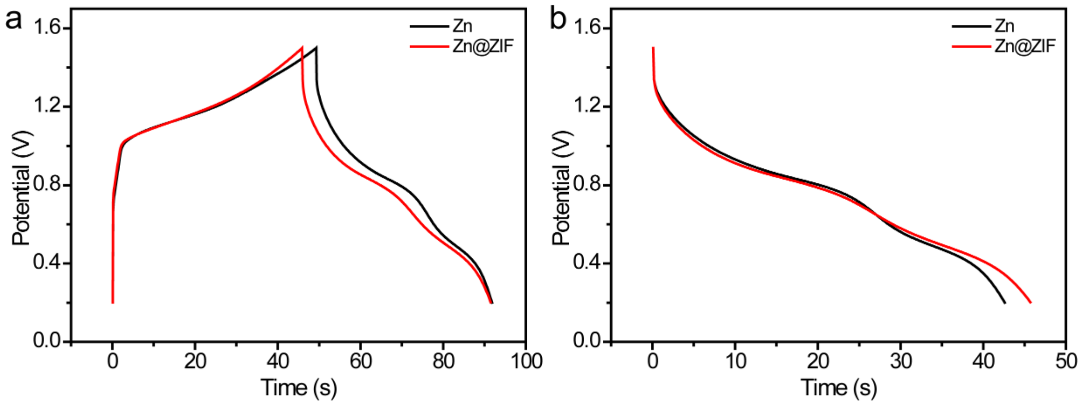

Figure S16. a) Charging-discharging profiles and b) discharge curves of the $LaVO_4$//Zn and $LaVO_4$//Zn-ZIF batteries at 10 mA cm^{-2}.

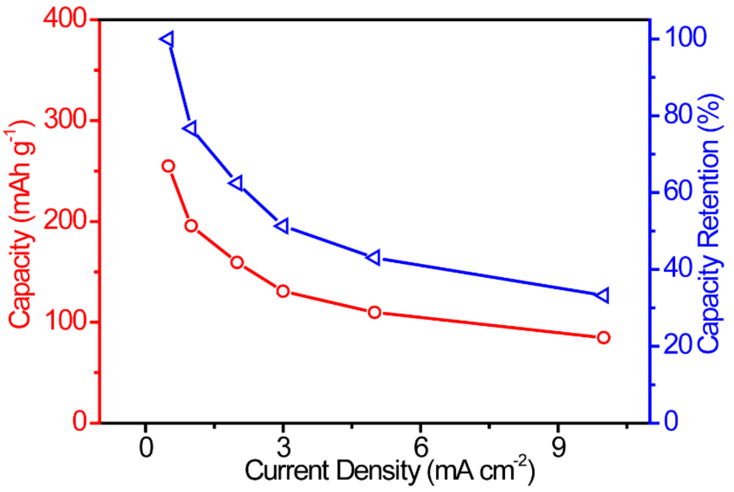

Figure S17. Capacities and capacity retention of the $LaVO_4$//Zn-ZIF battery at various current densities.
